# Facile room-temperature synthesis of carboxylated graphene oxide-copper sulfide nanocomposite with high photodegradation and disinfection activities under solar light irradiation

**DOI:** 10.1038/srep16369

**Published:** 2015-11-10

**Authors:** Shuyan Yu, Jincheng Liu, Wenyu Zhu, Zhong-Ting Hu, Teik-Thye Lim, Xiaoli Yan

**Affiliations:** 1 School of Civil and Environmental Engineering, Nanyang Technological University, 50 Nanyang Avenue, Singapore 639798, Republic of Singapore; 2 Faculty of Chemical Engineering and Light Industry, Guangdong University of Technology, Guangzhou, China 510009; 3 Nanyang Environment and Water Research Institute (NEWRI), Nanyang Technological University, 1 Cleantech Loop, CleanTech One, Singapore 637141, Republic of Singapore; 4 Environmental and Water Technology Centre of Innovation, 535 Clementi Road, Singapore 599489, Republic of Singapore

## Abstract

Carboxylic acid functionalized graphene oxide-copper (II) sulfide nanoparticle composite (GO-COOH-CuS) was prepared from carboxylated graphene oxide and copper precursor in dimethyl sulfoxide (DMSO) by a facile synthesis process at room temperature. The high-effective combination, the interaction between GO-COOH sheets and CuS nanoparticles, and the enhanced visible light absorption were confirmed by transmission electron microscopy (TEM), field emission scanning electron microscopy (FESEM), X-ray powder diffraction (XRD), Fourier transform infrared spectroscopy (FTIR), thermo gravimetric analysis (TGA), X-ray photoelectron spectroscopy (XPS), UV-vis diffuse reflectance spectra (DRS) and Photoluminescence (PL) spectra. The as-synthesized GO-COOH-CuS nanocomposite exhibited excellent photocatalytic degradation performance of phenol and rhodamine B, high antibacterial activity toward *E. coli* and *B. subtilis*, and good recovery and reusability. The influence of CuS content, the synergistic reaction between CuS and GO-COOH, and the charge-transfer mechanism were systematically investigated. The facile and low-energy synthesis process combined with the excellent degradation and antibacterial performance signify that the GO-COOH-CuS has a great potential for water treatment application.

The ever-growing demand for the clean water has stimulated significant research effort for the huge development of advanced water treatment technologies. Semiconductor-mediated heterogeneous photocatalysis is considered as a promising method to remove pathogens and organic pollutants from wastewater^1^. A great number of materials have been developed and involved, including Cu_2-x_S (0 ≤ x ≤), CdS, ZnO etc. Among those, copper sulfide (CuS) has been reported as one of the most important semiconductors due to its excellent chemical and physical properties at room temperature^2^. Various morphologies of CuS including nanopowders, nanotubes, nanorods, nanoribbons and so on have been successfully fabricated via versatile synthetic strategies such as solventless and solution thermolysis, solution-phase reaction, ultrasonic and microwave irradiation, etc^3^,^4^. However, those methods usually require complicated procedures and elevated temperatures, which greatly hinder the process of scale up. Recently, some relatively facile synthesis approaches have been advocated to prepare CuS-based semiconductor photocatalyst. For example, TiO_2_-CuS was obtained via a facile route using a sol-gel method by Park *et al.*^3^. Zhang *et al.* obtained CuS nanopowder by solvothermal method using Na_2_S as the sulfur source^4^. Nevertheless, there are still several issues limiting the application of CuS. To the best of our knowledge, there are few low-energy approaches developed for the synthesis of CuS-based composite photocatalyst, e.g., at room temperature. Moreover, the low photogenerated charge transfer rate on the photocatalyst surface and high aggregation of CuS nanomaterials, which significantly degrade the photocatalytic performance. An effective option for overcoming these drawbacks is to disperse the nanomaterials onto a suitable support, which could increase the surface area, inhibit the recombination of photogenerated charges, and allow easy recovery of the nanomaterials.

As a single layer of graphite, graphene has a perfect sp^2^-hybridized two-dimensional carbon structure, including perfect crystal and microstructure with large surface area^5^,^6^. Those properties can not only enhance photoactivity of other photocatalysts, but also make it be suitable support^7^. However, the van der Waals forces and high π-π stacking between adjacent layers, facilitate irreversible aggregation of graphene or even restacking to graphite, which greatly impede its application^8^. Along with a large number of different approaches that have been developed to address these obstacles, the most reliable and facile technique is the functionalization of graphene. Graphene oxide (GO) is an oxygen-containing graphene derivative with partial breakage of sp^2^–sp^2^ bonds into sp^3^–sp^3^ bonds for the insertion of some pendent groups like epoxy, carboxylic acid, and hydroxyl^9^,^10^,^11^. For example, with further carboxylation, GO-COOH has enhanced hydrophilicity and more carboxyl groups as anchoring sites to strongly bind other nanomaterials such as CuS to the surface of GO.

In this work, we put forward a facile yet efficient room-temperature synthesis approach for the fabrication of GO-COOH-CuS nanocomposite and explored its general environmental applications. A systematic characterization was carried out for the pristine materials (CuS and GO-COOH) and their composite (GO-COOH-CuS). Laboratory-scale batch experiments were conducted to investigate the photocatalytic degradation of phenol and rhodamine B (RhB), as well as the inactivation of *Escherichia coli* (*E. coli)* and *Bacillus subtilis* (*B. subtilis)* under solar light irradiation using the as-synthesized materials as the photocatalysts. A plausible charge-transfer mechanism is tentatively proposed for the photocatalysis process. To the best of our knowledge, the multifunctional GO-COOH-CuS nanocomposite is for the first time rapid synthesized via such facile and scalable room-temperature method and used for water decontamination and disinfection with excellent performance just during a short operating period under solar irradiation.

## Results and Discussion

### Morphological and structural analysis

**Figure 1:**
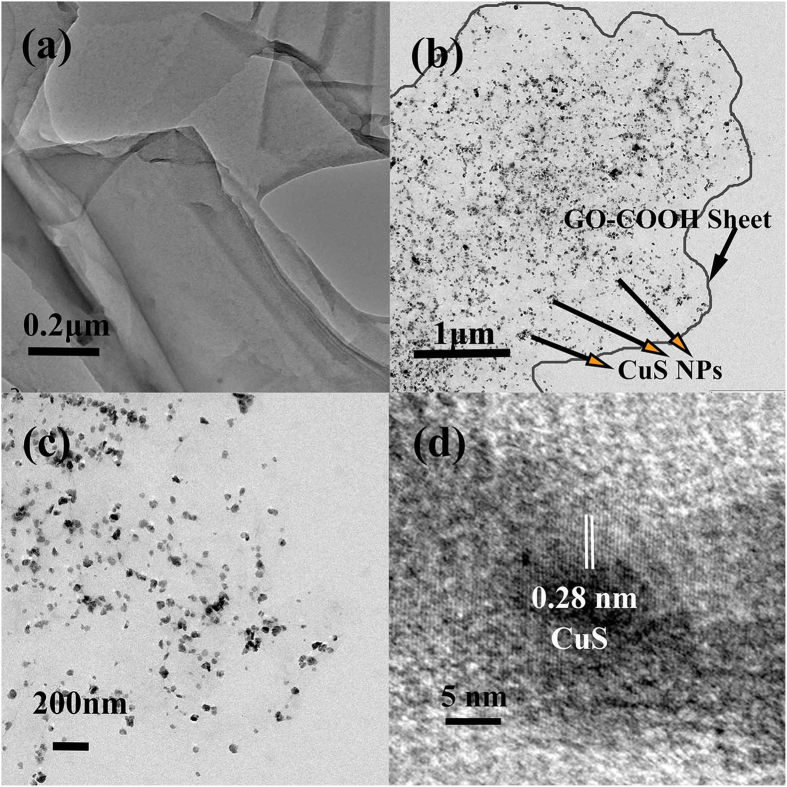
TEM images of (**a**) GO-COOH sheet, (**b–d**) GO-COOH-CuS nanocomposite.

TEM is employed to characterize the morphology and microstructure of the graphene-based catalyst, to verify the effective anchoring of CuS NPs onto the large GO-COOH sheets. Figure 1(a) presents that the size of the as-synthesized GO-COOH sheets is around 1–2 μm, which may benefit the recovery of GO-COOH based nanocomposite materials by simple filtration. The edge of GO-COOH sheets is clearly observed in Fig. 1(a–c), indicating no significant restacking of GO-COOH sheets. It may be due to the insertion of the carboxylic acid functional groups with CuS NPs anchored among layers. Moreover, as shown in Fig. 1(b,c), CuS NPs with sizes of 20–30 nm are highly monodispersed on the single GO-COOH sheet without any aggregation and no CuS NPs are observed outside the sheets. This can be attributed to the oxygen-containing groups, which provided uniformly anchoring sites on the both sides of GO-COOH sheets for CuS NPs. Thus the CuS NPs could anchor exactly on GO-COOH sheets and their aggregation was inhibited as well. The clear 0.28 nm lattice spacing of CuS NPs shown in Fig. 1(d) further confirms the effective self-anchoring of CuS NPs on the GO-COOH sheets. By the TEM analysis, it could be deduced that the carboxylic acid functional groups in the synthesis process have triple function: (1) increasing the hydrophilicity of GO sheets to facilitate CuS interaction with GO sheets; (2) reducing the van der Waals bond between carbon layers to promote the delamination of the GO sheets; and (3) providing strong anchoring sites for CuS NPs to inhibit their aggregation^12^.

**Figure 2:**
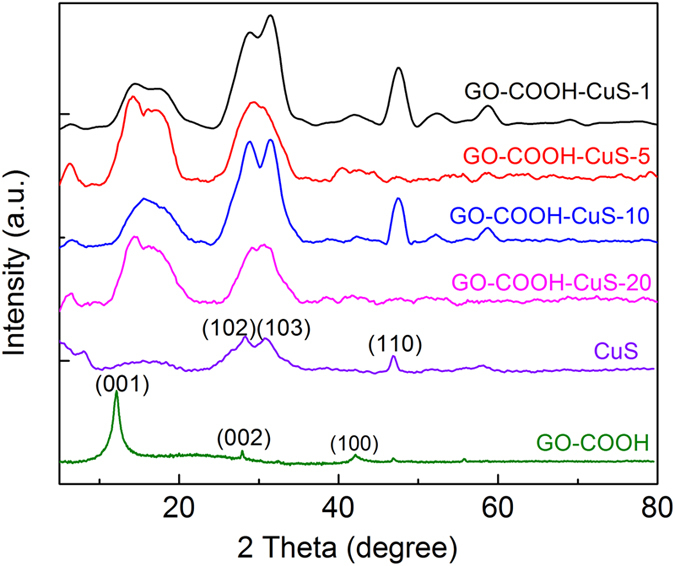
XRD patterns of CuS, GO-COOH and GO-COOH-CuS nanocomposites.

Figure 2 shows the XRD patterns of GO-COOH and GO-COOH-CuS. The observed diffraction peak at 2θ of 11.9° in GO-COOH can be attributed to the stacked GO-COOH sheet, which is consistent with the [001] interlayer spacing of 7.43 Ǻ^13^. In contrast, the [001] reflections of GO are not detected in the XRD patterns of all GO-COOH-CuS samples, indicating the regular stacks of GO were destroyed by the effective intercalation of CuS NPs. In addition, the obvious diffraction peaks at 29.76°, 33.85° and 48.30° can be assigned to (102), (103) and (110) facets of cubic CuS (JCPDS No. 01–1281), which further validate the effective anchoring of CuS NPs on the large GO-COOH sheets^14^.

**Figure 3:**
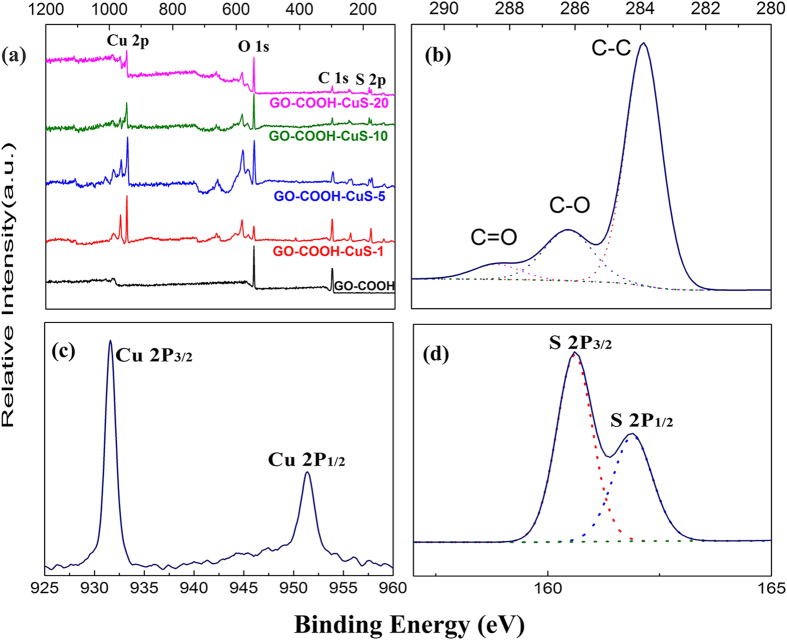
(**a**) Full XPS spectra of GO-COOH and of GO-COOH-CuS nanocomposites with different mass ratios; (**b–d**) high resolution XPS spectrum of C 1s, Cu 2p, S 2p of GO-COOH-CuS.

XPS is used to analyze the quantity of elements and the surface electronic state of the obtained products. The XPS spectra (Fig. 3) display graphitic C 1s, O 1s, Cu and S 2p peaks for GO-COOH-CuS nanocomposites, and the existence of C and O in the GO-COOH. In addition, the high-resolution C 1s spectra (Fig. 3b) exhibit three main separated peaks at 283.9 eV, 286.2 eV and 288.4 eV for all samples, which are connected to the graphitic C-C, C-O and COOH bonds, respectively. Figure 3c,d show the binding energies of Cu 2p_3/2_, Cu 2p_1/2_ peaks at 932.6 and 952.4 eV and double S 2P_3/2_, 2p_1/2_ peaks around 162 nm. The analysis of surface elemental composites of GO-COOH-CuS is reported in the Supporting Information (Supporting Table S1). The XPS results further confirm the carboxylation of GO and the existence of CuS in the composites, which is in good agreement with our subsequent TEM and XRD analyses.

### Composition and surface properties

**Figure 4:**
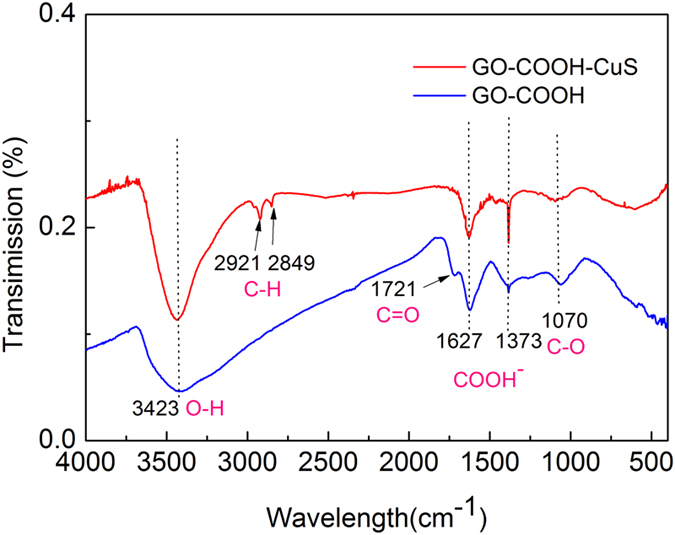
FTIR spectra of GO-COOH and GO-COOH-CuS samples.

The FTIR spectra of GO-COOH and GO-COOH-CuS are shown in Fig. 4. The bands at 1721 and 1070 cm^**−**1^ are ascribed to the C **= **O and C–O stretching vibrations of carboxylic acid group, respectively^15^. Furthermore, the doublet bands at 1627 and 1373 cm^**−**1^ correspond to the COO- symmetric and asymmertric vibrations complexed with surface Cu centers^16^. The doublet bands at 2921 and 2849 cm^**−**1^ in the GO-COOH-CuS FTIR spectrum are assigned to the C-H stretching vibrations^17^. The broad band ranging from 3000 to 3600 cm^−1^ with peak centered at 3423 cm^−1^ is attributed to the O–H stretching vibrations of adsorbed water molecules. The FTIR analysis confirmed the coordination between CuS and carboxylic acid groups in GO-COOH, which is essential and decisive for the controlling growth of CuS on the GO-COOH sheets.

**Figure 5:**
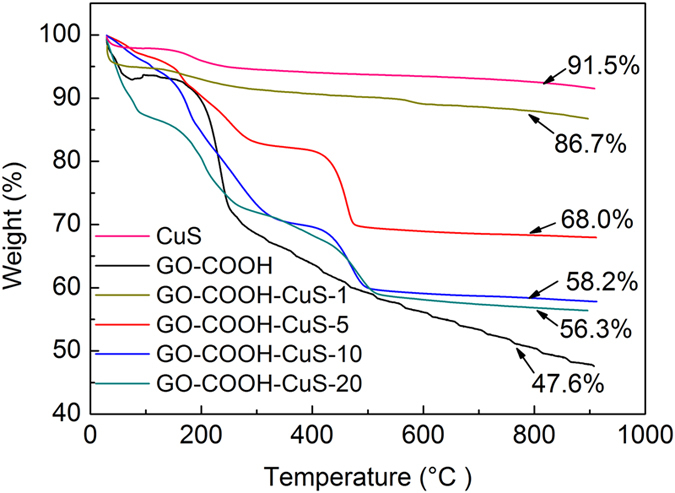
Thermogravimetric analysis of CuS, GO-COOH and GO-COOH-CuS nanocomposites.

TGA was used to evaluate the thermal stability of GO-COOH, CuS and GO-COOH-CuS. In Fig. 5, all materials show slight weight losses from 0 to 200 °C, which was due to the evaporation of tightly bounded water or some organic solvent molecules. In the case of GO-COOH, with the further increase of temperature, a sharp weight loss appears around 200 °C. It is resulted from the thermal decomposition of oxygen-containing functional groups^18^. After the temperature exceeds 200 °C, the weight of CO-COOH falls fair steadily. The grafting percentages of CuS on GO-COOH sheets could be estimated from the TGA thermograms. The mass percentage of CuS in the as-prepared composites of GO-COOH-CuS-1, −5, −10, −20 is about 89%, 47%, 24%, and 20%, respectively.

### Optical p**ro**perties

**Figure 6:**
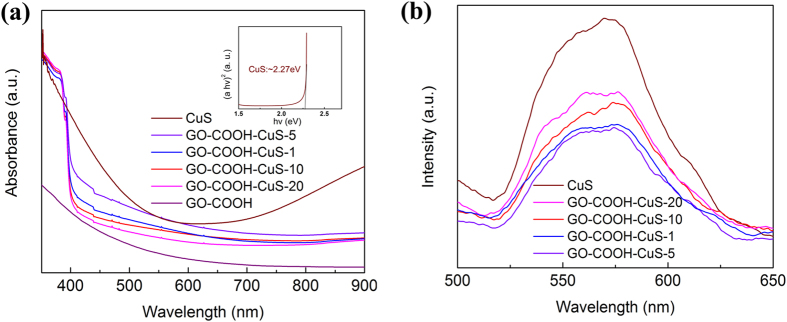
(**a**) UV-vis diffuse reflectance spectra (DRS) of CuS, GO-COOH and GO-COOH-CuS nanocomposites, and Kubelka-Munk transformed resflectance spectra of CuS (inset); (**b**) PL spectra (excitation at 400 nm) of CuS, GO-COOH and GO-COOH-CuS nanocomposites.

Figure 6(a) depicts the UV-vis diffuse reflectance spectra (DRS) of the CuS, GO-COOH and GO-COOH-CuS nanocomposite, as well as their Kubelka-Munk (K-M) transformed reflectance spectrum of CuS (inset). A notable broad absorption in the visible-light and near-infrared (NIR) region is observed from DRS for GO-COOH-CuS compared to GO-COOH, while E_g_ of CuS is 2.27 eV obtained by the formula ((ahν)^2^ **= **A(hν – *E*_*g*_))^19^. This suggests that the GO-COOH-CuS presents the enhanced absorbance over pure GO-COOH. The less amount of coupled CuS can effectively extend the absorption of GO-COOH onset to the visible light and NIR region, which is similar to the extended absorption for rGO-TiO_2_ composite caused by the formation of C-O-Ti bond^20^.

The effect of GO-COOH on the anti-recombination of e^−^/h^+^ was investigated by PL spectroscopy^21^. Figure 6(b) displays the as-synthesized nanomaterials with a broad emission peak at λ = 571 nm under an excitation at 400 nm. The GO-COOH-CuS nanocomposites exhibit a significantly lower PL emission intensity than pure CuS, which indicates that GO-COOH is effectively suppressing the e^−^/h^+^ recombination via faster electron transfer.

### Photocatalytic degradation of phenol **a**nd RhB

**Figure 7:**
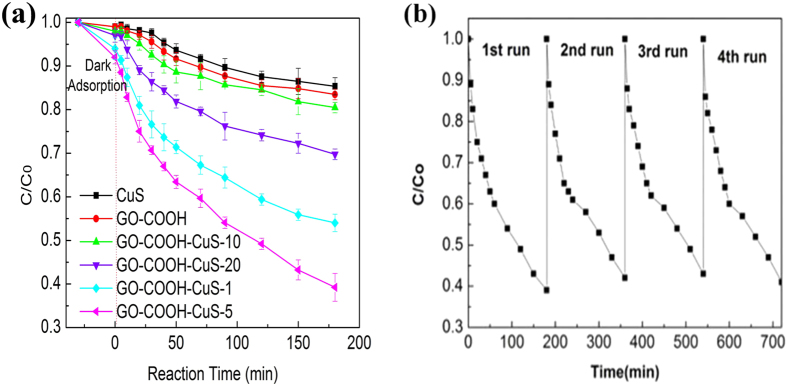
(**a**) Photocatalytic performance of CuS, GO-COOH and GO-COOH-CuS nanocomposites for phenol under solar light irradiation. (**b**) Four consecutive cycling curves of phenol photodegradation by GO-COOH-CuS-5.

**Figure 8:**
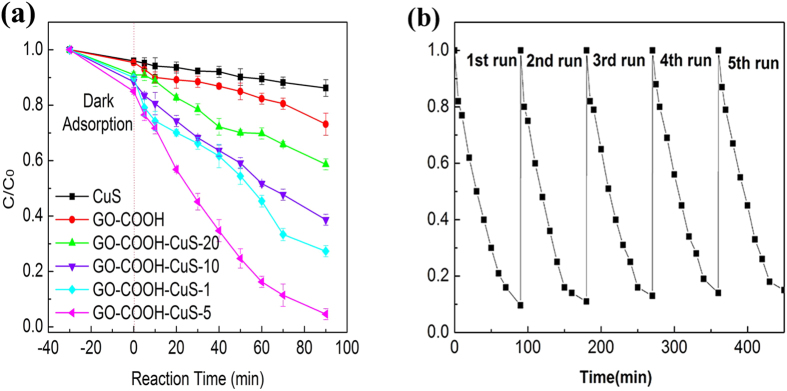
(**a**) Photocatalytic performance of CuS, GO-COOH and GO-COOH-CuS nanocomposites for RhB under solar light, (**b**) five consecutive cycling curves of RhB photodegradation by GO-COOH-CuS-5.

In order to explore their potential application in water treatment, phenol and RhB dye were taken as representative organic compounds to investigate the photodegradation ability of GO-COOH-CuS nanocomposites with various mass ratios (GO-COOH:CuS) under simulated solar light irradiation. Phenol is among the most resistant class of potential pollutant in the wastewater with strong toxicity and excellent solubility. RhB is a basic dye widely used and discharged by the paper printing, paint, textile dyeing and leather industries. Figures 7(a) and 8(a) show the concentration of pollutants as a function of degradation time. Compared to CuS and GO-COOH, GO-COOH-CuS nanocomposites exhibit higher adsorption for both phenol and RhB. Among the different mass ratios, GO-COOH-CuS-5 has the strongest adsorption ability, followed by GO-COOH-CuS-1, GO-COOH-CuS-10 and GO-COOH-CuS-20, as summarized in Table 1. The facilitation of the organic pollutants adsorption on nanocomposites may be due to their stronger non-covalent intermolecular forces. For example, the adsorption of phenol by GO-COOH sheets was caused by the interaction of π-conjugated and the ion-dipole interactions between GO-COOH and phenol due to the presence of similar conjugated aromatic cycles. This can also confirm the phenomenon that adsorption increases with the increasing graphene: CuS ratio, e.g., GO-COOH-CuS-5 > GO-COOH-CuS-1. However, it seems that π-conjugated and the ion-dipole interactions are limited for GO-COOH-10 and GO-COOH-20 because of excessive functional groups of GO-COOH, which causes a lower adsorption of phenol and RhB than GO-COOH-CuS-1 and GO-COOH-CuS-5.

As we known, the enhanced adsorption ability of nanomaterials could benefit the subsequent degradation process, which is confirmed in our work as well. In Figs 7a and 8a, the degradation ability of the nanocomposites for either phenol or RhB is coherent with their adsorption phenomenon. And in order to further investigate the long-term stability of synthesized GO-COOH-CuS composite, the photocatalytic degradation of phenol and RhB on GO-COOH-CuS-5 under simulated solar light irradiation was performed over five cycles. As shown in Figs 7b and 8b, no evident change was observed for the photocatalytic activity through the 5 cycles of experiments for both phenol and RhB degradation, indicating good photocatalytic stability of the GO-COOH-CuS composite.

**Figure 9:**
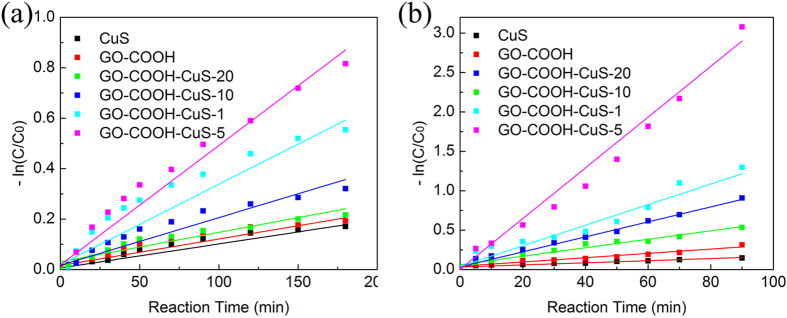
Pseudo first order photocatalytic degradation of (**a**) phenol and (**b**) RhB over the CuS, GO-COOH and GO-COOH-CuS nanocomposites.

In addition, Photocatalytic degradations for organic pollutants are often described in terms of the Langmuir-Hinshelwood model, which can be simplified as pseudo-first order reaction dC/dt **= **−kC, where *k* refers to the corresponding reaction rate kinetic constant. The photocatalytic degradation kinetic curves shown in Fig. 9 apparently follow the pseudo first-order kinetics in the studied concentration range. The calculated k (min^−1^) constants for all the applied nanomaterials are shown in Fig. 9 and the same order is found as degradation ability: GO-COOH-CuS-5 > GO-COOH-CuS-1 > GO-COOH-CuS-10 > GO-COOH-CuS-20 > GO-COOH > CuS (Table 1). Furthermore, the percentage of leached Cu^2+^ after degradation followed the same order as summarized in Table 1. All these results indicate that integration of CuS with GO-COOH sheets could enhance the photocatalytic activity, due to the improved light absorption, increased pollutants adsorption and inhibition of charge combination, etc. But the optimal mass ratio is preferred for the best performance of nanocomposites, such as GO-COOH-CuS-5 in this study. This could be explained as following: (1) For the GO-COOH-CuS-5, the well combination of GO-COOH and CuS nanoparticle can motivate more effective charge transfer from conduction band (CB) of CuS to GO-COOH, to achieve low recombination rates of photogenerated electron-hole pairs; (2) the GO-COOH-CuS-10 and GO-COOH-CuS-20 with small amount of CuS has limited capabilities to absorb solar light, which results in poor photodegradation abilities; and (3) the GO-COOH-CuS-1 with great amount of CuS also shows lower ability than GO-COOH-CuS-5 because the excessive CuS NPs will cover most of the reactive sites on GO-COOH surface and also act as recombination centers, leading to the decreased photocatalytic ability^22^.

**Figure 10:**
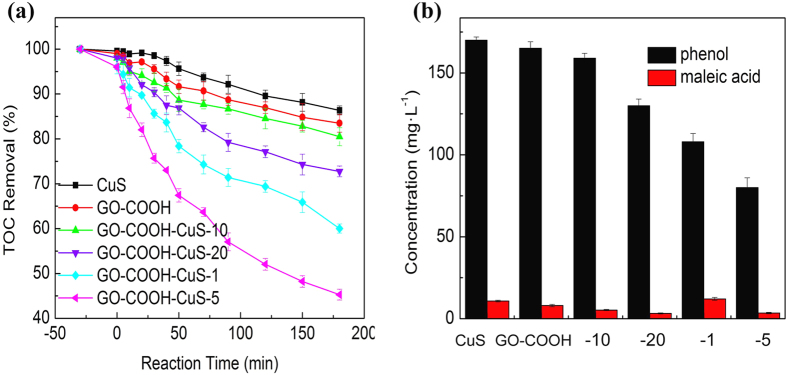
(**a**) TOC concentrations versus time, and (**b**) HPLC analyses of byproducts of phenol degradation on CuS, GO-COOH and GO-COOH-CuS nanocomposites under 3 h solar light irradiation.

The total organic content (TOC) analysis was monitored as a function of the rate of mineralization of phenol and its related intermediates. As the results shown in Table 1 and Fig. 10(a), the TOC was decreasing over time, and the GO-COOH-CuS-5 displayed the highest TOC removal ability. Moreover, the main intermediates of phenol degradation were analyzed by HPLC. After 3 h solar irradiation, the main intermediate formed was identified as maleic acid, which is non-toxic and at low concentration. Those toxic intermediates such as catechol and benzoquinone were not found, which may due to their further degradation during 3 h reaction.

### Disinfection performance

**Figure 11:**
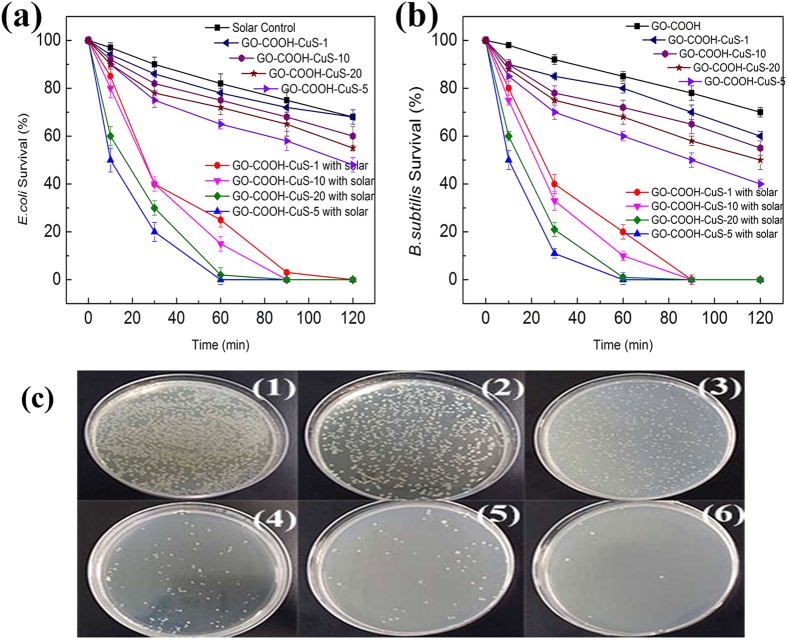
Antibacterial activities of GO-COOH-CuS nanocomposites with and without solar irradiation (**a**) *E.coli* survival, (**b**) *B. subtilis* survival, and (**c**) agar plates pictures of *E.coli* exposed to GO-COOH-CuS-5 under solar light with time (0 min, 10 min, 30 min, 60 min, 90 min and 120 min).

The antibacterial activity of GO-COOH and GO-COOH-CuS nanocomposites was evaluated with or without simulated solar light irradiation, using both Gram-negative *E. coli* and Gram-positive *B. subtilis* bacteria as the bacterium models. As the results shown in Fig. 11, the nanomaterials could inactive both bacterial strains in the darkness and showed higher antibacterial activity with CuS anchoring, indicating CuS NPs play an important role in the toxicity. It may due to the release of Cu^2+^ ions. A possible mechanism is that Cu^2+^ can form chelates with biomolecules or dislodge in some metalloproteins which may cause their dysfunction and further cell inactivation^23^. However, it cannot infer that the more CuS content, the higher toxicity of nanocomposites because the excess CuS may cover the adsorption sites of GO-COOH sheets, leading to the decreased attachment of bacteria on the surface of nanocomposites. In this work, GO-COOH-CuS-5 exhibits the highest toxicity due to its optimal coupling ratio. After 60 min exposure to GO-COOH-CuS-5, nearly 40% *E. coli* and 30% *B. subtilis* were inactivated.

Under solar light irradiation, both bacterial strains exposed to the nanocomposites had a much lower survival rate contrast to that in darkness. The improved disinfection efficiency is attributable to the synergistic effect in photocatalysis process. GO-COOH sheets adsorb bacteria on the surface, providing more biocontact opportunities for CuS NPs. Aside from the toxicity of the released Cu^2+^, the photoinduced reactive oxygen species (ROS) by CuS NPs can attack the attached bacteria as well, leading to the enhanced antibacterial activity. Among those nanocomposites, GO-COOH-CuS-5 again shows the highest photocatalytic disinfection efficiency due to its optimal ratio of coupling content. It inactivated 90% *E. coli* and 80% *B. subtilis* after 30 min reaction. With the further increase of contact time to 60 min, the *E. coli* and *B. subtilis* were inactivated nearly by 100%. The higher inactivation efficiency to *B. subtilis* indicates that Gram-positive bacteria were more susceptible to the photodynamic effects than Gram-negative bacteria, which might be due to resistance of the outer membrane in Gram-negative bacteria. An additional outer membrane of Gram-negative bacteria can protect their inner layer from radical or chemical attacking^24^.

### 2.6 Photocatalytic mechanism

**Figure 12:**
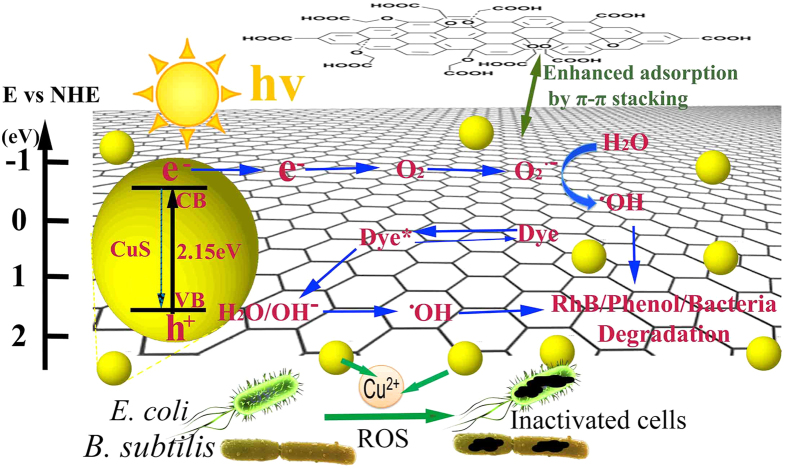
Schematic illustration of proposed photocatalytic and antibacterial mechanisms of GO-COOH-CuS nanocomposite.

For the enhancement of photocatalytic activity, an efficient charge transfer is crucial^25^. In the system of GO/PcZn, GO/TiO_2_ and rGO/CuS, GO has shown excellent property to be an effective acceptor and transporter, suppressing the recombination of electron-hole pairs and facilitating charge transfer^20^,^26^,^27^. Similarly, a charge transfer might be achieved in our GO-COOH-CuS nanocomposite system, where GO-COOH sheets act as the electron acceptor. Based on the experimental results and analyses, a possible mechanism for the enhanced photocatalytic activity and good stability of synthesized GO-COOH-CuS nanocomposite is proposed in Fig. 12. Under solar light irradiation, the electrons could be generated either from excited dye (RhB*) molecules or CuS NPs, then transfer to GO-COOH. They react with absorbed surface O_2_ on GO-COOH sheets to produce reactive oxygen species (ROS) which assist the pollutants degradation. Meanwhile, the photogenerated holes on CuS also could oxidize pollutants. In addition to the charge separation, the photocatalytic activity can be influenced by the adsorption of pollutants, the light absorption and the surface oxygen adsorption as well. The enhanced visible light absorption of GO-COOH-CuS is verified by UV-vis absorption analysis (Fig. 5). The high adsorption ability of GO-GOOH sheets for organic pollutants has been confirmed by previous works^21^,^28^, as well as in our study (Figs 7 and 8). All these improved properties of GO-COOH-CuS result in the higher photocatalytic activity towards our synthesized nano-system.

## Materials and Methods

### Materials

Sodium nitrate (NaNO_3_, 99%), potassium permanganate (KMnO_4_, 99%), hydrogen peroxide (H_2_O_2_, 35%), concentrated sulfuric acid (H_2_SO_4_, 98%), chloroacetic acid (ClCH_2_CO_2_H, 99%), concentrated hydrochloric acid (HCl, 36.5%), and thioacetamide (C_2_H_5_NS, 99%) were purchased from Sigma-Aldrich (Singapore). Copper (II) nitrate trihydrate (Cu(NO_3_)_2_, 99.5%) was purchased from Strem Chemicals. Natural graphite (SP1) was purchased from the Bay Carbon Company (USA). Ethanol, acetone, dimethyl sulfoxide (DMSO), and rhodamine B (RhB) were purchased from Merck Ltd (Singapore). The deionized (DI) water was produced from Millipore Milli-Q water purification system.

### Synthesis of GO-COOH sheets and GO-COOH-CuS nanocomposites

GO sheets were exfoliated from natural graphite according to the modified Hummers’ method^12^,^29^. GO-COOH was synthesized under strongly basic condition to convert hydroxyl groups to carboxylic acid moieties by activating the GO sample with chloroacetic acid (ClCH_2_CO_2_H)^12^. In a typical process, 1.2 g of chloroacetic acid, 1 g of NaOH and 50 mg of GO were added to 100 mL DI water and then sonicated for 3 h to speed up the elimination of sodium chloride. The resulting GO-COOH solution was neutralized by HNO_3_, and purified with acetone and water for several times.

**Figure 13:**
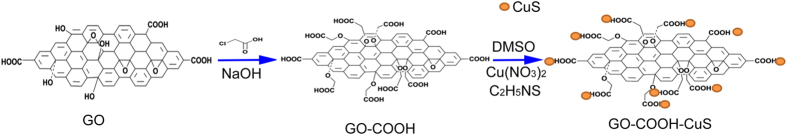
Synthetic route of GO-COOH and GO-COOH-CuS nanocomposites.

For the synthesis of GO-COOH-CuS nanocomposite, we herein developed a facile and scalable method to anchor CuS nanoparticles (NPs) on large GO-COOH sheets at room temperature. Briefly, various amounts (1 mL, 5 mL, 10 mL and 20 mL) of 1 mg mL^−1^ GO-COOH solution were mixed with 1 mmol of Cu(NO_3_)_2_, 1 mmol of C_2_H_5_NS and 10 mL of DMSO. DMSO acted as a solvent and co-reactant to furnish the sulfur source in the form of H_2_S. The mixture was stirred for 24 h at ambient temperature to ensure CuS NPs anchored on GO-COOH sheets. The products were centrifuged and rinsed with acetone, ethanol and DI water several times to remove the residual. The resulting solid samples were freeze-dried for 2 h under −50 °C to obtain the final GO-COOH-CuS catalysts. Here, the samples prepared by addition of various amounts GO-COOH solutions (1 mL, 5 mL, 10 mL and 20 mL) were named as GO-COOH-CuS-1, GO-COOH-CuS-5, GO-COOH-CuS-10 and GO-COOH-CuS-20, respectively. The synthesis process is illustrated in Fig. 13.

### Characterizations

X-ray powder diffraction (XRD) patterns were taken on a D8-Advance Bruker-AXS diffractometer using Cu K_α_ irradiation to determine the crystallinity phase. Transmission electron microscopy (TEM) images were carried out using a JEOL 2010-H microscope TEM operating at 200 kV. The samples were prepared for the analysis by dropping dilute solutions of samples on 400-mesh carbon-coated copper grids and leading the solvent to dry. Fourier transform infrared spectra (FTIR) were collected on a Bruker FTIR spectroscopy (Nicolet IS10) with solid powder samples. Thermogravimetric analysis (TGA) was recorded using a NETESCH STA 409 PC thermgravimeter. Briefly, 5–6 mg samples were heated from room temperature to 900 °C in dry nitrogen at a rate of 10 °C min^−1^. X-ray photoelectron spectroscopy (XPS) measurements were taken by using a Kratos Axis Ultra Spectrometer with a 15 kV and 10 mA monochromic Al K_α_ source at 1486.7 eV. The UV–vis diffuse reflectance spectra (DRS) of the 0.1 mg mL^−1^ dispersion solutions were measured by using an evolution 300 spectrophotometer under room temperature. The photoluminescence (PL) emission spectra were recorded on a fluorescence spectrometer (PerkinElmer). The leaching of Cu^2+^ was checked by quantification of Cu^2+^ concentration in solution using micro plasma atomic emission spectroscopy (MP 4100 AES). The total organic carbon (TOC) was measured using a TOC analyzer (TOC-L CPH/CPN; Shimadzu, Kyoto, Japan). The analysis of intermediates of phenol degradation was conducted by a high performance liquid chromatography (HPLC, Agilent 1100) with a reverse-phase column (Agilent, Eclipse XDB-C18, 150 × 4.6 mm).

### Photocatalytic degradation of phenol and RhB under solar irradiation

In order to investigate the application potential for wastewater treatment, the photocatalytic degradation of phenol and RhB using the as-synthesized nanocomposites were conducted in a laboratory-scale batch photoreactor with 8 quartz capsules. A 500 W Xenon lamp was used as the simulated solar light source. Cool deionized water was circulated through a quartz jacket. And the distance between the light source and the quartz test tubes contained our samples was 15 cm where the measured light intensity was 100 mW cm^−2^. Before commencement of irradiation, the reaction suspension containing organic pollutants (40 mg L^−1^ RhB or 200 mg L^−1^ phenol) and photocatalysts (1 g L^−1^) were stirred in the dark for 1 h to achieve equilibrium. Samples of 3 mL solution were withdrawn from the quartz test tubes and centrifuged to collect supernatants. The concentrations of RhB and phenol were analyzed by recording their maximum absorbance on the UV-Vis spectrophotometer at the wavelengths of 550 nm and 270 nm, respectively.

### Photocatalytic disinfection under solar irradiation

Gram-negative bacteria *E. coli* (ATCC 8739) and Gram-positive bacteria *B. subtilis* (ATCC 6633) were chosen as standard organisms to evaluate the antibacterial ability of GO-COOH-CuS. Normally, *E. coli* and *B. subtilis* were cultivated in Luria-Bertani nutrient solution followed by incubation at 37 °C and 30 °C respectively for 18 h to get the exponential growth phase. The pure *E. coli* and *B. subtilis* cells were harvested by centrifugation and washed with saline solution (0.9% NaCl) to remove residual macromolecules. Then, the cells were re-suspended in the saline solution to maintain a concentration of ~10^8^ colony forming units (cfu mL^−1^). All glass apparatuses and solutions used in the experiments were sterilized in autoclave at 121 °C for 20 min to ensure sterility.

The photocatalytic disinfection experiments were performed in the photochemical reactor. The nanocomposites (GO-COOH-CuS-1, GO-COOH-CuS-5, GO-COOH-CuS-10 and GO-COOH-CuS-20) were magnetically mixed with 10^8^ cfu mL^−1^ *E. coli* or *B. subtilis* saline solution. After irradiation under simulated solar light for 2 h, 100 μL of solution was daubed uniformly and cultivated on the solidified agar nutrient plates. The colony forming units were counted and compared with control plates to calculate ratio of cell viability (*C*/*C*_*0*_). As comparison, a contrast experiment under dark condition and a blank control experiment without any nanomaterial were carried out under the same condition. All the experiments were conducted in triplicates.

## Conclusions

In summary, with a facile and up scalable synthesis process, multifunctional GO-COOH-CuS nanocomposites were successfully synthesized. The uniform deposition of CuS NPs on GO-COOH sheets contributes to the superior photocatalytic activities of the nanocomposite, not only towards organic pollutants (phenol and RhB) but also waterborne pathogens (*E. coli* and *B. subtilis*). Herein, GO-COOH was demonstrated as an appropriate conductive ligand to support the CuS NPs used in photocatalysis. In addition, as the electron acceptor, GO-COOH improved the lifetime of electron-hole pairs of CuS resulting in a better pathway for electron transport. The GO-COOH-CuS nanocomposite material can overcome the limitations of the pristine GO-COOH and CuS photocatalysts, and combine the merits of the both materials to maximum synergistic effects, including (1) perfect contact between GO-COOH sheets and CuS NPs to minimize the photocorrosion. (2) uniformly distributed CuS NPs onto the GO-COOH surface to improve isolating photogenerated electrons and holes, meanwhile, to enhance the charge transfer. Moreover, the durability study showed that the photocatalytic activity of the GO-COOH-CuS is stable enough for multiple recycling. This study signifies that GO-COOH-CuS nanocomposite can be a promising photocatalyst in the field of water decontamination and disinfection.

## Additional Information

**How to cite this article**: Yu, S. *et al.* Facile room-temperature synthesis of carboxylated graphene oxide-copper sulfide nanocomposite with high photodegradation and disinfection activities under solar light irradiation. *Sci. Rep.* **5**, 16369; doi: 10.1038/srep16369 (2015).

## Supplementary Material

Supplementary Information

## Figures and Tables

**Table 1:** Adsorption percentage (%), photo degradation efficiencies (%) and kinetics (min^−1^) of all as-synthesized samples.

	GO-COOH	CuS	GO-COOH-CuS			
			−1	−5	−10	−20
Adsorption (%)						
* Phenol*	2.3	1.4	6.3	8.5	4.2	3.7
* RhB*	6.1	5.9	12.2	15.1	12.8	9.3
Photo degradation (%)						
* Phenol*	16.4	15.7	46.2	61.8	30.2	20.1
* TOC removal*	15.6	14.7	40.0	55.2	29.3	19.6
* RhB*	13.8	26.8	72.7	95.4	61.3	41.3
Kinetics (min^−1^)						
* Phenol*	0.0011	0.00095	0.0032	0.0047	0.0019	0.0012
* RhB*	0.0027	0.0014	0.0013	0.032	0.0095	0.0054
Leached Cu^2+^ after photodegradation(%)						
* Phenol*	/	19.5	6.4	5.8	9.5	10.1
* RhB*	/	15.2	4.5	3.4	7.8	9.3
