# Supplementary material for: Facile room-temperature synthesis of carboxylated graphene oxide-copper sulfide nanocomposite with high photodegradation and disinfection activities under solar light irradiation
Source: Sci Rep. 2015 Nov 10;5:16369. doi: 10.1038/srep16369 (PMC4639843; doi:10.1038/srep16369)
Supplement: Supplementary Information [file srep16369-s1.pdf]

Supporting Information

Facile room-temperature synthesis of carboxylated graphene oxide-copper sulfide nanocomposite with high photodegradation and disinfection activities under solar light irradiation

Shuyan Yu[1], Jincheng Liu[1,2*], Wenyu Zhu[1], Zhong-Ting Hu[1], Teik-Thye Lim[1,3], Xiaoli Yan[1,3*]

1. School of Civil and Environmental Engineering, Nanyang Technological University, 50 Nanyang Avenue, Singapore 639798, Republic of Singapore

2. Faculty of Chemical Engineering and Light Industry, Guangdong University of Technology, Guangzhou, China 510009

3. Nanyang Environment and Water Research Institute (NEWRI), Nanyang Technological University, 1 Cleantech Loop, CleanTech One, Singapore 637141, Republic of Singapore

Corresponding authors: Dr. Jincheng Liu, jcliu@gdut.edu.cn; Prof. Xiaoli Yan, XLYAN@ntu.edu.sg, Tel: +65-67906934.

SUPPORTING INFORMATION

2 Data Analyses

1 Table

SI-1 HPLC Analyses Report

Analyses of phenol and its degradation intermediates were carried out by the high performance liquid chromatography (HPLC, Agilent 1100) with a reverse-phase column (Agilent, Eclipse XDB-C18, 150 × 4.6 mm). The mobile phase was composed of acetonitrile and deionized and doubly distilled water of a v/v ratio at 10 : 90. The absorbance detector was set at 270 nm. The flow rate was 1 mL/min.

SI-1.1, Supporting data S1,

HPLC analyses of mixtures of 100 mg $\cdot L^{-1}$ catechol, maleic acid, oxalic acid and phenol as standard solution

Specific Retention Time(t_R):

1.611min: oxalic acid

1.690 min: maleic acid

5.965min: phenol

9.225min: catechol

<Chromatogram>

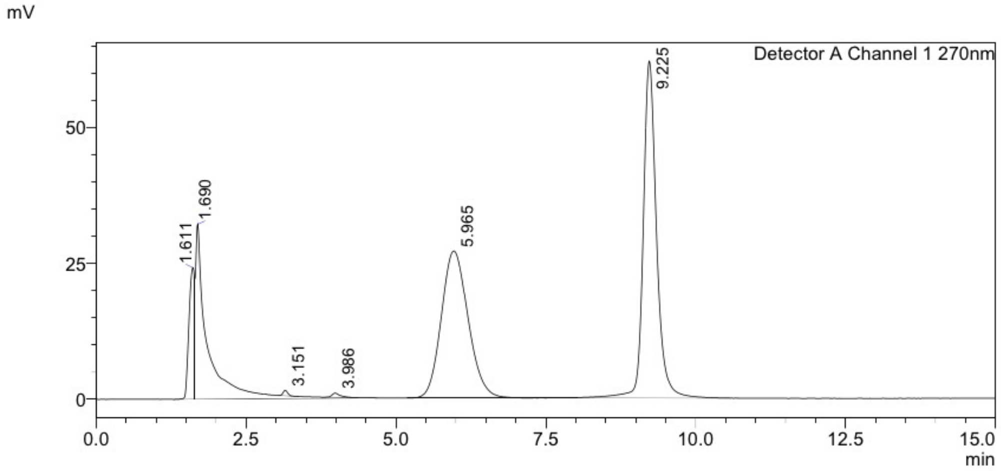

<Peak Table> Detector Channel 270nm

Peak	Ret. Time	Area	Height	Conc. ($mg\cdot L^{1}$)
**1**	1.611	157134	24182	100.0
**2**	1.690	449623	32189	100.0

**3**	3.151	5272	998	Un-identified
**4**	3.986	7549	773	Un-identified
**5**	5.965	846452	27052	100.0
**6**	9.225	965173	61946	100.0
**Total**		2431202	147140	

SI-1.2, Supporting data S2,

HPLC analysis of byproducts of phenol degradation on GO-COOH-CuS-10 nanocomposite under 3 h solar light irradiation.

Specific Retention Time(t_R):

1.391min: one non-toxic small molecule organic

1.685 min: maleic acid

5.947min: phenol

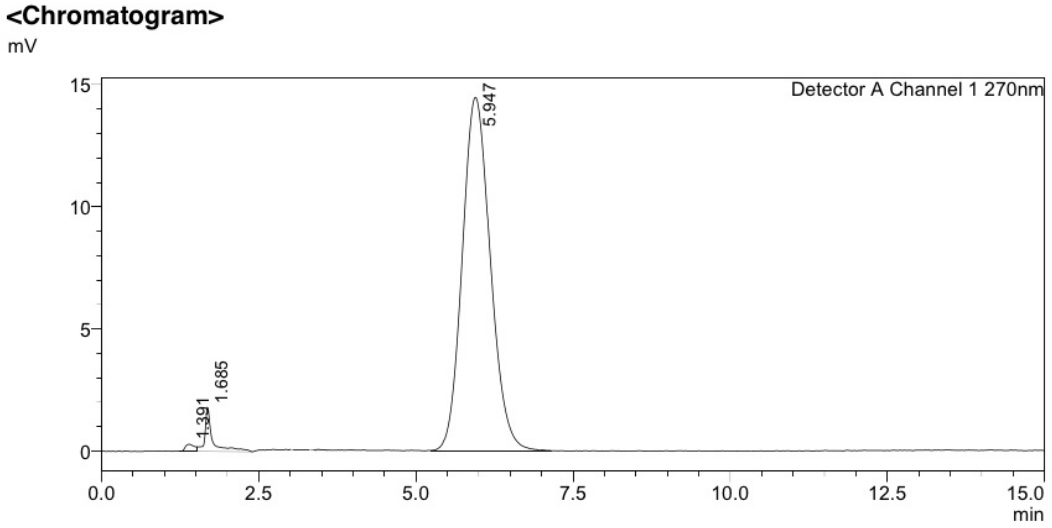

<Peak Table> Detector Channel 270nm

Peak	Ret. Time	Area	Height	Conc. (mg·L^{1})
**1**	1.391	2991	295	Un-identified
**2**	1.685	15269	1794	5.3
**3**	5.947	450312	14462	159.4
**Total**		468571	16552	

SI-2 XPS Analysis Report

Table S1. Elemental composition of GO-COOH-CuS

Element （At%)	GO-COOH-CuS				
	GO-COOH	-1	-5	-10	-20
**Cu**	0	8.01	8.11	10.96	1.09
S	1.43	24.76	19.38	21.97	12.99
**O**	28.39	38.96	13.40	38.51	28.39
**C**	70.18	28.27	52.50	28.55	54.60
